# Procedures between training and reactivation influence the destabilization of instrumental sucrose memory

**DOI:** 10.3389/fnbeh.2022.953629

**Published:** 2022-09-14

**Authors:** Chaoran Cheng, Marc T. J. Exton-McGuinness, Jonathan L. C. Lee

**Affiliations:** School of Psychology, University of Birmingham, Birmingham, United Kingdom

**Keywords:** instrumental (operant) behavior, reconsolidation boundaries, destabilization, MK-801, ratio schedules of reinforcement, memory reconsolidation

## Abstract

Memory destabilization and reconsolidation is hypothesized to be a fundamental mnemonic process that can underpin memory updating. Instrumental memories have been shown recently to be destabilized following a reactivation session that involves a change in instrumental reward contingency. However, the acquisition and performance of an instrumental response occurs in the presence of the learning of other reward-related memories. This may influence the ability of a given reactivation session to destabilize the previously learned instrumental memory. Here we present a series of experiments in male rats involving an instrumental memory trained on an FR1 schedule over 10 days, and then reactivated in a session that imposed a VR5 schedule of reinforcement. When MK-801 was injected prior to the VR5 reactivation session, it reliably impaired subsequent instrumental performance at test only when the reactivation session occurred 48 h, and not 24 h, after the end of training. The interposition between the end of training and the reactivation session of a context extinction session, an additional VR5 reactivation session, or indeed the simple experience of being handled and injected with vehicle, resulted in MK-801 no longer having an amnestic effect on test performance. While we do not have a clear account for the process and mechanism underpinning this apparent selectivity of the effect of the VR5 session to destabilize the instrumental memory, it does additionally highlight the need for greater understanding of the conditions that facilitate reactivation-induced memory destabilization.

## Introduction

The memories underpinning instrumental responding have recently been shown to undergo reconsolidation. Memory reconsolidation involves a memory reminder triggering the destabilization of the previously learned memory, necessitating a process of memory restabilization (Haubrich and Nader, 2018). Therefore, the destabilization of a memory is evidenced typically by successful disruption of the restabilization process, resulting in reduced performance at test. While some studies have shown that instrumental memory destabilizes following non-reinforced instrumental responding (Tedesco et al., 2014; Piva et al., 2019, 2020), others show consistently that brief extinction training was insufficient to trigger instrumental memory destabilization (Hernandez and Kelley, 2004; Mierzejewski et al., 2009; Exton-McGuinness et al., 2014). Instead, a change in instrumental contingency seemingly reliably resulted in memory destabilization (Exton-McGuinness et al., 2014, 2019; Exton-McGuinness and Lee, 2015).

For well-learned instrumental sucrose memories, a substantial change in instrumental contingency was employed to trigger memory destabilization (Exton-McGuinness et al., 2014), whereas a lesser change was used to destabilize a more weakly learned instrumental memory (Exton-McGuinness and Lee, 2015). This comparison is consistent with the apparent parametric relationship between the strength of initial learning and the requirements of the reactivation session to destabilize the memory (Suzuki et al., 2004; Reichelt and Lee, 2012a), which might relate to the necessity for a sufficient prediction error signal to trigger memory destabilization (Reichelt and Lee, 2012b). However, such a viewpoint does not take into consideration the multiple memory representations that are formed in any learning experience, and the relative impact that the reactivation session has on each representation.

In an instrumental learning setting, reinforced lever pressing not only leads to instrumental action-outcome and stimulus-response associations, but also pavlovian associations between the reinforcer and discrete or contextual stimuli (Milton and Everitt, 2010). While action-outcome and stimulus-response associations may be mutually inhibitory, thereby allowing only one of the two to control behavior (Bouton, 2021), instrumental and pavlovian associations may be active in parallel. This leads to the question of whether two independent associations can both be destabilized in parallel by the same reactivation session.

In the pavlovian memory literature, it appears that when there are two competing memory traces, it is the one that controls behavior that is impaired by amnestic treatment (Eisenberg et al., 2003). That is, a memory appears to destabilize when it is dominant at the time of memory reactivation. However, this relationship has been observed exclusively in the setting of reconsolidation competing with extinction (Eisenberg et al., 2003; Suzuki et al., 2004; Merlo et al., 2014). Nevertheless, it raises the possibility that the potentially competing relationship between instrumental and pavlovian memories might influence destabilization of each. Indeed, in studies of cue-supported instrumental cocaine seeking the pavlovian and instrumental memories have each been impaired (Lee et al., 2006; Exton-McGuinness et al., 2017). However, these observations have resulted from distinct reactivation parameters and a single intervention has yet to be successful in impacting upon both pavlovian and instrumental memories.

Even in the absence of discrete pavlovian stimuli, the change of instrumental contingency that is sufficient for instrumental memory destabilization also causes a substantial degradation of the pavlovian context-reward memory, in that the density of reinforcement while present in the operant context is greatly reduced. Therefore, we originally hypothesized that this reduction in the strength of the context-reward memory was permissive for instrumental memory destabilization.

In our established instrumental memory reconsolidation setting, here we used a VR5 schedule of reinforcement, instead of the VR20 employed previously (Exton-McGuinness et al., 2014). Initially, we predicted that this VR5 reactivation session would in itself be insufficient to destabilize the instrumental memory due to the more limited contingency change from FR1 at training. However, we hypothesized that interposing a context extinction session between instrumental training and memory reactivation might reduce the influence of the context-reward memory and thereby reduce the magnitude of the contingency change necessary to destabilize the instrumental memory. Indeed, if there were a competition between different memory traces such that only one can be destabilized at a time, it might be that the VR20 reactivation preferentially destabilizes the instrumental memory, whereas a VR5 reactivation preferentially destabilizes the context-reward memory. We observed, however, that the simple interposing of a day with no behavioral session more reliably facilitated subsequent memory destabilization.

## Materials and methods

### Subjects

Subjects were 256 experimentally naïve male Hooded-Lister rats (Charles River, United Kingdom) weighing 200–250 g (median 225 g) at the beginning of the experiment. Rats were kept in a conventional animal facility on a 12 h light/dark cycle (lights on 0700), housed in quads in cages containing aspen chip bedding. Environmental enrichment was available in the form of a Plexiglass tunnel. Water was provided *ad libitum*. The rats were food restricted and fed 80 g/cage/day chow from the first day of behavioral training. Experimental sessions took place 1200–1600 each day. At the end of the experiment, all animals were humanely killed *via* a rising concentration of CO_2_. All procedures were approved by a local ethical review board and carried out in accordance with the United Kingdom Animals (Scientific Procedures) Act 1986, Amendment Regulations 2012 (PPL P8B15DC34 and PPL P3B19B9D2).

### Drugs

MK-801 (AbCam, United Kingdom) was dissolved in sterile saline to a concentration of 0.1 mg/ml. Rats were injected i.p. with 0.1 mg/kg of MK-801 (Exton-McGuinness et al., 2014; Exton-McGuinness and Lee, 2015) or 1.0 ml/kg saline vehicle. This dose has been demonstrated to disrupt instrumental memory reconsolidation. Memantine (Tocris, United Kingdom) was dissolved in sterile saline with 8% DMSO to a concentration of 20 mg/ml. Rats were injected i.p. with 20 mg/kg of memantine (Sachser et al., 2016) or 1.0 ml/kg phosphate-buffered saline. All injections were assigned systematically by cage, randomly within each cage.

### Behavioral procedures

Behavioral sessions took place in eight operant chambers (MedAssociates, Fairfax, VT, USA), as described previously (Exton-McGuinness and Lee, 2015).

#### Training

All rats were trained for ten sessions. In each session, one or two levers (depending on the experiment) were extended into the operant chamber. In the 2-lever experiments, one lever was assigned pseudo-randomly to be the active lever, counterbalanced across groups. The training session started with illumination of the house light and the insertion of the lever(s). When the active lever was depressed, a 45-mg sucrose pellet was delivered into the food magazine (FR1 schedule). The lever did not retract and no explicit stimulus was paired with the reward. No programmed consequences occurred if the inactive lever was depressed. The training sessions lasted for 30 min, or until the maximum of 60 sucrose pellets was obtained. Only one training session was given to each rat per day for a total 10 days. Training took place on weekdays only.

#### Reactivation

All experimental groups received a reactivation session. The reactivation session was similar to the training session, except reinforcement occurred under a VR5 schedule. VR5 required a random number of active lever presses to gain sucrose reward (mean: 5, range: 1–9). Reactivation lasted 20 min, or until the maximum of 20 pellets was obtained. Rats were injected with MK-801 or saline 30 min before the reactivation session, unless otherwise indicated:

• In the direct reactivation condition, the VR5 reactivation session took place 24 h after the last training session.
• In the delayed reactivation condition, the VR5 reactivation session took place 48 h after last training session.
• In the context extinction condition, a 30-min exposure to the training context alone (houselight illuminated and levers retracted through the session) took place 24 h after the last training session. The VR5 reactivation session took place a further 24 h later (48 h after the last training session).
• In the delayed injection condition, the VR5 reactivation session took place 48 h after last training session, but MK-801 or saline was injected 6 h after the reactivation session.
• In the double reactivation experiment, there were two VR5 reactivation sessions, which took place 24 and 48 h after last training session.

**FIGURE 1:**
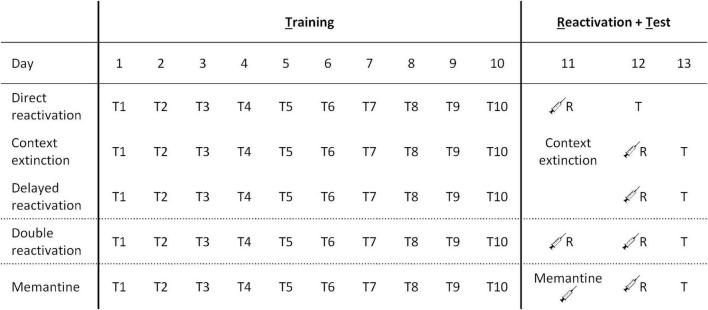
Behavioral procedures across different experiments. The three reactivation procedures employed involved direct reactivation (VR5 reactivation on the day after the 10th day of training), context extinction (context extinction and FR5 reactivation sessions on consecutive days after the 10th day of training), and delayed reactivation (VR5 reactivation 2 days after the 10th day of training). In other experiments, the VR5 reactivation was repeated on two consecutive days (double reactivation), or there was an injection of memantine (or PBS vehicle) on the intervening day within the delayed reactivation condition.

See Figure 1 for a comparative overview of the different procedures.

#### Test

All groups received a test session 24 h following the reactivation session (or the second reactivation session in the double reactivation experiment). The levers were extended and the house light illuminated during the session. However, no sucrose pellet was delivered when any lever was pressed. The test session lasted for 30 min.

### Statistical analyses

Data are presented as mean ± S.E.M. and were analyzed using SPSS 27. Data were analyzed by factorial ANOVA, with α = 0.05 and $\eta_{p}^{2}$ reported as an index of effect size. For the analysis of the training sessions, Session was included as a factor; where appropriate a Greenhouse–Geisser correction was applied to correct for sphericity violations. The primary outcome of interest was the lever responding in 1-lever experiments and discriminated active (vs. inactive) lever responding in the 2-lever experiments. Therefore, the primary statistical outcome of interest was the MK-801 × condition interaction (and planned analysis of the simple main effect of MK-801) in the 1-lever experiments, and the MK-801 × condition × lever interaction (and planned analysis of the MK-801 × lever interaction in each condition) in the 2-lever experiments. The one lever results were also subjected to a mini-meta-analysis (*Meta-essentials*).

## Results

### Destabilization of instrumental memory in a 1-lever setting

**FIGURE 2:**
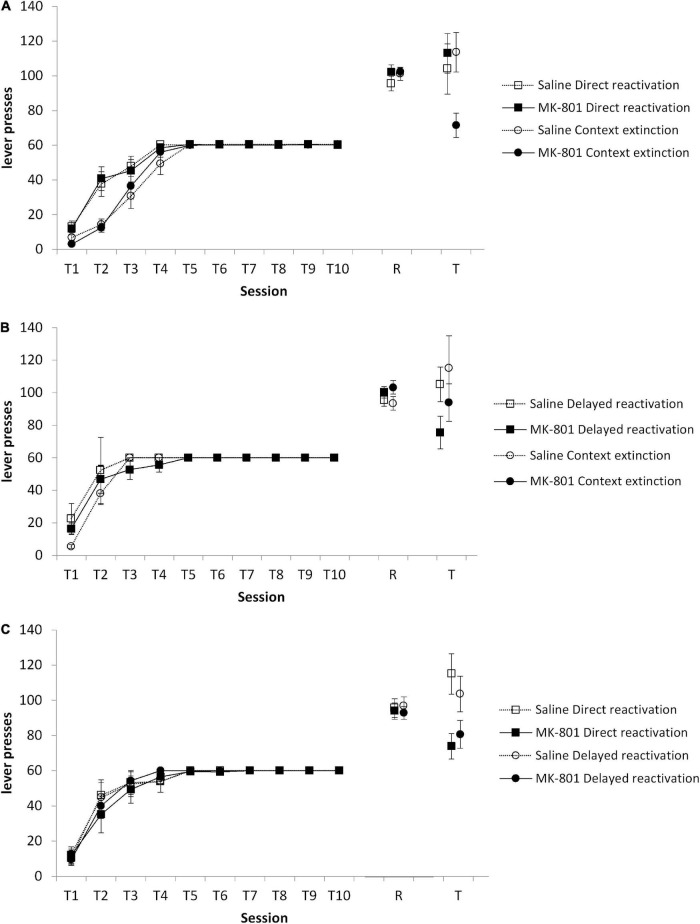
Varied effects of MK-801 in context extinction, direct reactivation, and delayed reactivation conditions. Rats were trained to press a single lever for sucrose reward across 10 days (T1–T10) and then received a VR5 reactivation session (R) following MK-801 or Saline injection. **(A)** Lever pressing at test (T) was impaired in the context extinction condition, but not in the direct reactivation condition. In the context extinction condition previously MK-801 treated rats (*n* = 12) showed significantly impaired lever pressing compared to Saline-treated control (*n* = 13). No differences were observed during training or at reactivation. In the direct reactivation condition, there was no obvious difference between MK-801 (*n* = 12) and saline (*n* = 12) treated rats across any phase of the experiment. **(B)** Lever pressing at test was impaired in the delayed reactivation condition, but not in the context extinction condition. In the delayed reactivation condition previously MK-801 treated rats (*n* = 7) showed significantly impaired discriminated lever pressing compared to Saline-treated controls (*n* = 7). No differences were observed during training or at reactivation. In the context extinction condition, there was no obvious difference between MK-801 (*n* = 8) and saline (*n* = 8) treated rats across any phase of the experiment. **(C)** Lever pressing at test was impaired in the direct reactivation condition, but not in the delayed reactivation condition. In the direct reactivation condition previously MK-801 treated rats (*n* = 8) showed significantly impaired discriminated lever pressing compared to Saline-treated control (*n* = 7). No differences were observed during training or at reactivation. In the delayed reactivation condition, there was no obvious difference between MK-801 (*n* = 8) and saline (*n* = 8) treated rats across any phase of the experiment. Data are presented as mean ± SEM.

We conducted a series of pairwise reactivation comparisons to evaluate the efficacy of context extinction to facilitate the subsequent destabilization of instrumental memory by a VR5 reactivation session in a 1-lever instrumental setting. Initially, context extinction was compared against a group that proceeded directly from training to VR5 reactivation on the next day. At test, there was no Condition × MK-801 interaction [Figure 2A; *F*(1,45) = 1.59, *p* = 0.214, $\eta_{p}^{2}$ = 0.034], with no overall effect of MK-801 [*F*(1,45) = 2.39, *p* = 0.129, $\eta_{p}^{2}$ = 0.050]. Planned comparisons revealed an effect of MK-801 treatment to reduce responding in the context extinction [*F*(1,22) = 9.10, *p* = 0.006, $\eta_{p}^{2}$ = 0.214], but not the direct reactivation condition [*F*(1,21) = 0.24, *p* = 0.094, $\eta_{p}^{2}$ < 0.001]. These differences at test were not evident at the reactivation session [Condition × MK-801: *F*(1,45) = 0.53, *p* = 0.472, $\eta_{p}^{2}$ = 0.012; MK-801: *F*(1,45) = 1.09, *p* = 0.303, $\eta_{p}^{2}$ = 0.024; context extinction condition: *F*(1,23) = 0.067, *p* = 0.900, $\eta_{p}^{2}$ = 0.003; direct reactivation condition: *F*(1,22) = 1.23, *p* = 0.279, $\eta_{p}^{2}$ = 0.053], or during training [Condition × MK-801: *F*(1,45) = 0.001, *p* = 0.973, $\eta_{p}^{2}$ < 0.001; MK-801: *F*(1,45) = 0.016, *p* = 0.900, $\eta_{p}^{2}$ < 0.001; context extinction condition: *F*(1,23) = 0.017, *p* = 0.899, $\eta_{p}^{2}$ = 0.001; direct reactivation condition: *F*(1,22) = 0.003, *p* = 0.954, $\eta_{p}^{2}$ < 0.001]. Therefore, interposition of a context extinction session between training and VR5 reactivation appeared to facilitate instrumental memory destabilization. A separate experiment indicated that implementing the 30-min context exposure prior to the start of instrumental training (rather than afterward as in the context extinction condition) was not successful in facilitating instrumental memory destabilization (see Supplementary material).

Given that the training to reactivation interval is different between the context extinction and direct reactivation conditions, we then compared context extinction to a delayed reactivation condition, in which rats had a day with no behavioral session before VR5 reactivation. Surprisingly, at test, there was evidence that VR5 reactivation was more effective following a delay than following context extinction (Figure 2B). Although there was no Condition × MK-801 interaction [*F*(1,26) = 0.10, *p* = 0.760, $\eta_{p}^{2}$ = 0.004] and no overall effect of MK-801 [*F*(1,26) = 3.57, *p* = 0.070, $\eta_{p}^{2}$ = 0.121], planned comparisons revealed an effect of MK-801 treatment to reduce responding in the delayed reactivation [*F*(1,12) = 6.22, *p* = 0.028, $\eta_{p}^{2}$ = 0.341], but not the context extinction condition [*F*(1,14) = 0.86, *p* = 0.368, $\eta_{p}^{2}$ = 0.058]. These differences at test were not evident at the reactivation session [Condition × MK-801: *F*(1,26) = 0.22, *p* = 0.643, $\eta_{p}^{2}$ = 0.008; MK-801: *F*(1,26) = 3.89, *p* = 0.059, $\eta_{p}^{2}$ = 0.130; delayed reactivation condition: *F*(1,12) = 1.30, *p* = 0.277, $\eta_{p}^{2}$ = 0.341; context extinction condition: *F*(1,14) = 2.75, *p* = 0.119, $\eta_{p}^{2}$ = 0.164], or during training [Condition × MK-801: *F*(1,26) = 0.023, *p* = 0.881, $\eta_{p}^{2}$ = 0.001; MK-801: *F*(1,26) = 0.027, *p* = 0.870, $\eta_{p}^{2}$ = 0.001; delayed reactivation condition: *F*(1,12) < 0.001, *p* = 0.994, $\eta_{p}^{2}$ < 0.001; context extinction condition: *F*(1,14) = 0.09, *p* = 0.796, $\eta_{p}^{2}$ = 0.006]. Therefore, it may be that the training-reactivation interval is the important factor in facilitating VR5-induced instrumental memory destabilization.

In order to test directly the training-reactivation interval, we compared the delayed and direct reactivation conditions in a further cohort of rats. At test, there was an overall effect of MK-801 [Figure 2C; *F*(1,27) = 11.45, *p* = 0.002, $\eta_{p}^{2}$ = 0.298], with no Condition × MK-801 interaction [*F*(1,27) = 0.92, *p* = 0.347, $\eta_{p}^{2}$ = 0.033]. Planned comparisons revealed an effect of MK-801 treatment to reduce responding in the direct reactivation [*F*(1,13) = 8.62, *p* = 0.012, $\eta_{p}^{2}$ = 0.399], but not the delayed reactivation condition [*F*(1,14) = 3.22, *p* = 0.094, $\eta_{p}^{2}$ = 0.187]. These differences at test were not evident at the reactivation session [Condition × MK-801: *F*(1,27) = 0.06, *p* = 0.812, $\eta_{p}^{2}$ = 0.002; MK-801: *F*(1,27) = 0.38, *p* = 0.544, $\eta_{p}^{2}$ = 0.014; direct reactivation condition: *F*(1,13) = 0.06, *p* = 0.808, $\eta_{p}^{2}$ = 0.005; delayed reactivation condition: *F*(1,14) = 0.42, *p* = 0.528, $\eta_{p}^{2}$ = 0.029], or during training [Condition × MK-801: *F*(1,27) = 0.09, *p* = 0.768, $\eta_{p}^{2}$ = 0.003; MK-801: *F*(1,27) = 0.08, *p* = 0.777, $\eta_{p}^{2}$ = 0.003; direct reactivation condition: *F*(1,13) = 0.14, *p* = 0.716, $\eta_{p}^{2}$ = 0.011; delayed reactivation condition: *F*(1,14) < 0.001, *p* = 0.992, $\eta_{p}^{2}$ < 0.001]. These results suggested that, across the three experiments, none of the behavioral procedures resulted in a consistent destabilization of instrumental memory.

Given that we had two replications of each condition across the three experiments, as well as not observing a Condition × MK-801 interaction in any of the experiments, we conducted a subgroup mini-meta-analysis on our data (Goh et al., 2016). The overall meta-analysis had a statistically significant overall outcome of MK-801 to reduce responding at test (*Z*-value = −3.10, *p* < 0.001), with moderate heterogeneity (*Q* = 9.71, *I*^2^ = 48.51%). Subgroup analysis revealed that the delayed reactivation condition was the only condition with a homogenous population (*Q* = 0.05, *I*^2^ = 0.00%; direct reactivation: *Q* = 5.12, *I*^2^ = 80.48%; context extinction: *Q* = 1.47, *I*^2^ = 31.84%), with an effect size (Hedges’ g) of −1.16 (95% CI −0.99 to −1.33). Therefore, implementing a delay between training and VR5 reactivation was the most reliable method to induce instrumental memory destabilization within this 1-lever setting.

Finally, analysis of session lengths during instrumental training and VR5 reactivation revealed little evidence that the groups differed in their learning and performance of the instrumental response (see Supplementary material).

### Destabilization of instrumental memory in a 2-lever setting

In order to establish the generality of our findings, we conducted a follow-up series of experiments using a 2-lever instrumental paradigm. We also included a 6-h delayed injection control, which is commonly used to strengthen the conclusion that it is treatment within the reconsolidation window that results in subsequent performance impairment.

**FIGURE 3:**
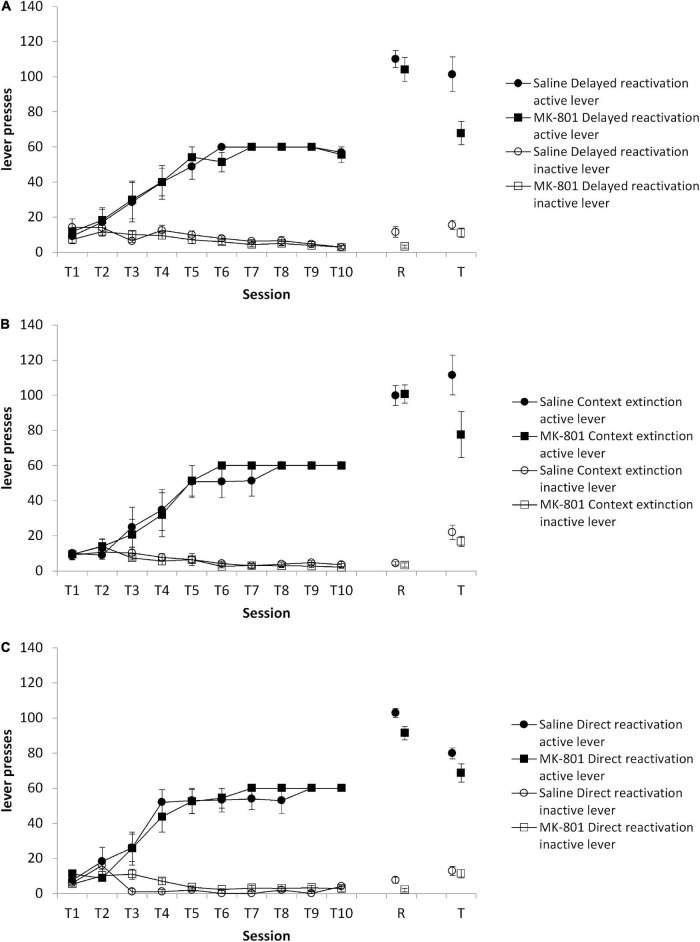
Discriminated lever pressing at test (T) was impaired in the delayed reactivation condition, but not the context extinction and direct reactivation conditions. Rats were trained to press an active lever for sucrose reward across 10 days (T1–T10) and then received a VR5 reactivation session (R) following MK-801 or Saline injection. **(A)** In the delayed reactivation condition previously MK-801 treated rats (*n* = 7) showed significantly impaired discriminated lever pressing compared to Saline-treated control (*n* = 7). No differences were observed during training or at reactivation. **(B)** In the context extinction condition, there was no obvious difference between MK-801 (*n* = 5) and saline (*n* = 6) treated rats across any phase of the experiment. **(C)** In the direct reactivation condition, there was also no obvious difference between MK-801 (*n* = 8) and saline (*n* = 8) injected rats. Data are presented as mean ± SEM.

First, in a comparison of the three reactivation conditions at test, there was an overall MK-801 × lever interaction [Figure 3; *F*(1,33) = 9.44, *p* = 0.004, $\eta_{p}^{2}$ = 0.222], but no Condition × MK-801 × lever interaction [*F*(2,33) = 1.37, *p* = 0.267, $\eta_{p}^{2}$ = 0.077]. Planned comparisons revealed an effect of MK-801 to impair discriminated lever responding in the delayed reactivation condition [Figure 3A; MK-801 × lever: *F*(1,12) = 6.25, *p* = 0.028, $\eta_{p}^{2}$ = 0.343], but not in the context extinction [Figure 3B; MK-801 × lever: *F*(1,10) = 3.40, *p* = 0.095, $\eta_{p}^{2}$ = 0.254] and direct reactivation [Figure 3C; MK-801 × lever: *F*(1,11) = 0.46, *p* = 0.511, $\eta_{p}^{2}$ = 0.040] conditions. These differences at test were not evident at the reactivation session [Condition × MK-801 × lever: *F*(2,33) = 0.310, *p* = 0.736, $\eta_{p}^{2}$ = 0.018; Condition × MK-801: *F*(2,33) = 1.174, *p* = 0.32, $\eta_{p}^{2}$ = 0.066; direct reactivation MK-801 × lever: *F*(1,12) = 0.054, *p* = 0.820, $\eta_{p}^{2}$ = 0.004; delayed reactivation MK-801 × lever: *F*(1,11) = 0.838, *p* = 0.380, $\eta_{p}^{2}$ = 0.004; context extinction MK-801 × lever: *F*(1,10) = 0.067, *p* = 0.801, $\eta_{p}^{2}$ = 0.071], or during training [Condition × MK-801 × lever: *F*(2,104.8) = 0.009, *p* = 0.991, $\eta_{p}^{2}$ = 0.001; Condition × MK-801: *F*(2,33) = 0.315, *p* = 0.732, $\eta_{p}^{2}$ = 0.019: direct reactivation MK-801 × lever: *F*(1,31.5) = 0.193, *p* = 0.669, $\eta_{p}^{2}$ = 0.017; delayed reactivation MK-801 × lever: *F*(1,30.3) = 0.124, *p* = 0.731, $\eta_{p}^{2}$ = 0.010; context extinction MK-801 × lever: *F*(1,26.1) = 0.199, *p* = 0.665, $\eta_{p}^{2}$ = 0.020]. Therefore, there was greater evidence for an effect of MK-801 in the delayed reactivation condition than other reactivation conditions.

A comparison of the same delayed reactivation group against the control delayed injection group similarly revealed an overall MK-801 × lever interaction at test [*F*(1,27) = 9.081, *p* = 0.006, $\eta_{p}^{2}$ = 0.252], but no Condition × MK-801 × lever interaction [*F*(1,27) = 0.944, *p* = 0.340, $\eta_{p}^{2}$ = 0.034]. Planned comparisons confirmed a lack of effect of MK-801 on discriminated responding in the delayed injection condition [MK-801 × lever: *F*(1,15) = 1.68, *p* = 0.215, $\eta_{p}^{2}$ = 0.101; saline: active lever = 117.2 ± 8.1, inactive lever = 14.8 ± 1.4; MK-801: active lever = 101.8 ± 8.7, inactive lever = 13.4 ± 1.0]. There was no difference between the delayed reactivation and delayed injection groups at the reactivation session [Condition × MK-801 × lever: *F*(1,30) = 1.297, *p* = 0.264, $\eta_{p}^{2}$ = 0.041; Condition × MK-801: *F*(1,30) = 0.065, *p* = 0.800, $\eta_{p}^{2}$ = 0.002; delayed injection MK-801 × lever: *F*(1,18) = 2.406, *p* = 0.138, $\eta_{p}^{2}$ = 0.118; data not shown]. Nor were there differences during training [Condition × MK-801 × lever: *F* (1,30) = 0.012, *p* = 0.914, $\eta_{p}^{2}$ < 0.001, Condition × MK-801: *F*(1,30) = 0.096, *p* = 0.759, $\eta_{p}^{2}$ = 0.003; delayed injection MK-801 × lever: *F*(1,18) = 0.053, *p* = 0.821, $\eta_{p}^{2}$ = 0.003; data not shown].

### Repeated VR5 reactivation

**FIGURE 4:**
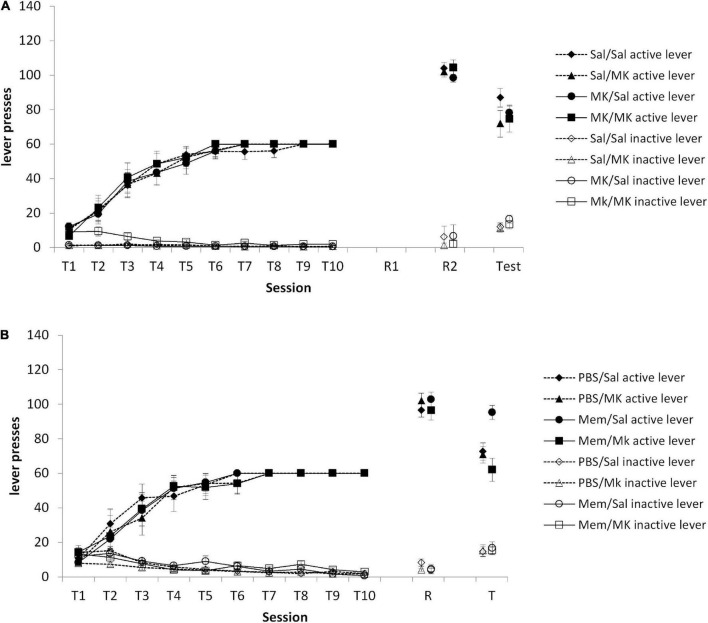
**(A)** No obvious impact of MK-801 when administered prior to either or both of two consecutive reactivation sessions. Rats were trained to press an active lever for sucrose reward across 10 days (T1–T10) and then received two VR5 reactivation sessions (R1 and R2) following MK-801 or Saline injection. There was no obvious difference between MK/MK (*n* = 12), MK/Sal (*n* = 14), Sal/MK (*n* = 14), and Sal/Sal (*n* = 13) treated rats across any phase of the experiment. **(B)** Discriminated lever pressing at test (T) was impaired in the memantine group, but the PBS controls. Rats were trained to press an active lever for sucrose reward across 10 days (T1–T10) and received an Memantine or PBS injection 24 h after training, then received one VR5 reactivation session (R) following MK-801 or Saline injection. In the memantine injected groups, MK-801 treated rats (*n* = 8) showed significantly impaired discriminated lever pressing compared to Saline-treated control (*n* = 8). In the PBS injected groups, there was also no obvious difference between MK-801 (*n* = 8) and saline (*n* = 8) injected rats. Data are presented as mean ± SEM.

As we originally hypothesized that a VR5 session might preferentially destabilize a non-instrumental memory trace (e.g., context-reward), we conducted an experiment in which VR5 sessions were performed on consecutive days, with injections of MK-801 (or saline) prior to each session. The prediction was that if a VR5 session on the day after training destabilized the context-reward memory, its reconsolidation should be impaired by MK-801 such that the subsequent VR5 session should destabilize the instrumental memory. At test, there was no overall Injection1 × Injection2 × lever interaction [Figure 4A; *F*(1,46) = 1.21, *p* = 0.277, $\eta_{p}^{2}$ = 0.026]. Planned comparisons of the Injection2 × lever interaction within each Injection1 group (Saline vs. MK-801) confirmed a lack of effect of MK-801 at the second VR5 reactivation [Saline: *F*(1,22) = 1.86, *p* = 0.187, $\eta_{p}^{2}$ = 0.078; MK-801: *F*(1,24) = 0.003, *p* = 0.955, $\eta_{p}^{2}$ < 0.001]. Therefore, contrary to our prediction, the performance of a VR5 session, regardless of drug treatment, rendered the second VR5 session ineffective at rendering test behavior vulnerable to the impact of MK-801.

### Memantine treatment between training and reactivation

Our results suggest that some process takes place in the 24–48 h after training that facilitates the destabilization of the instrumental memory by a VR5 reactivation session. One possibility is the involvement of ongoing plasticity processes after the end of training. For example, active forgetting occurs after learning in a manner that is disrupted by memantine (Sachser et al., 2016), and cellular mechanisms of consolidation can be engaged for up to 20 h after learning (Arguello et al., 2013). Therefore, we injected memantine or vehicle on the intervening day between training and reactivation, predicting that this would prevent the VR5 session from destabilizing the instrumental memory. At test, there was a significant Memantine × MK-801 × lever interaction [Figure 4B; *F*(1,26) = 7.45, *p* = 0.011, $\eta_{p}^{2}$ = 0.223]. Planned comparisons revealed an effect of MK-801 in the memantine-treated rats [MK-801 × lever: *F*(1,13) = 14.59, *p* = 0.002, $\eta_{p}^{2}$ = 0.529], but not in the vehicle-injected rats [MK-801 × lever: *F* (1,13) = 0.58, *p* = 0.462, $\eta_{p}^{2}$ = 0.042]. These differences at test were not evident at the reactivation session [Memantine × MK-801 × lever: *F*(1,28) = 2.62 *p* = 0.116, $\eta_{p}^{2}$ = 0.086; MK-801 × lever in memantine-treated rats: *F*(1,14) = 0.59 *p* = 0.455, $\eta_{p}^{2}$ = 0.040; MK-801 × lever in vehicle-injected rats: *F*(1,14) = 3.77 *p* = 0.073, $\eta_{p}^{2}$ = 0.212] or during training [Memantine × MK-801 × lever: *F*(1,28) < 0.001 *p* = 0.997, $\eta_{p}^{2}$ < 0.001; MK-801 × lever in memantine-treated rats: *F*(1,14) = 0.002 *p* = 0.970, $\eta_{p}^{2}$ < 0.001; MK-801 × lever in vehicle-injected rats: *F*(1,14) = 0.001 *p* = 0.976, $\eta_{p}^{2}$ < 0.001].

## Discussion

Here, we have shown that across 1-lever and 2-lever instrumental learning settings, a reactivation procedure that involves exposure to a VR5 session 2 days after the final of 10 days of training most reliably resulted in an effect of pre-VR5 injection of MK-801 to impair instrumental performance at a subsequent test. This was compared against reactivation procedures that presented the VR5 session the day after the end of training, or with the interposition of a context extinction session. When rats received two consecutive VR5 reactivation sessions on the days following the end of training, there was little evidence of an effect of MK-801 when injected prior to either or both sessions. Finally, while a simple injection of saline on the day between the end of training and the VR5 reactivation appeared to render pre-VR5 MK-801 ineffective, an injection of memantine had no such effect.

Across a number of experiments and conditions, we observed cases in which the injection of MK-801 prior to a VR5 reactivation session resulted in impaired instrumental performance at a subsequent test. We have previously made similar observations in both sucrose and cocaine instrumental lever pressing settings (Exton-McGuinness et al., 2014, 2019; Exton-McGuinness and Lee, 2015), the interpretation of which has been that of an impairment in the reconsolidation of the instrumental sucrose or cocaine memory. Here, the observation that pre-VR5 MK-801 does not result in test impairment across all our conditions indicates that in the cases where we do observe an impairment, it is unlikely to be a result of long-term non-specific effects of MK-801 on instrumental performance. The strongest evidence for this is perhaps from the mini meta-analysis of our 1-lever experiments. While we do not have a non-reactivation control condition in the 1-lever setting, the mini meta-analysis provides an alternative means of support for the conclusion that MK-801 impairs subsequent instrumental performance only when the VR5 reactivation session is delayed 2 days after the end of training. This reactivation-dependence provides the same interpretative value as the non-reactivation control that is typically necessary to conclude reconsolidation impairments (Dudai, 2004). In our 2-lever experiments, independent analyses of the different conditions in our planned comparisons support the conclusion that MK-801 only has a disruptive impact when injected prior to, and not 6 h after, a delayed VR5 reactivation session. However, we did not observe strict statistical injection time-dependence of the effects of MK-801. Nevertheless, our results are consistent with an impairment in the reconsolidation of the instrumental sucrose memory.

That the VR5 reactivation session reliably destabilized the instrumental sucrose memory when implemented 2 days after the end of training (as opposed to 1 day or after a context extinction session) has also been observed in an instrumental cocaine seeking setting (Exton-McGuinness et al., 2019). It is, however, not immediately obvious why the extra 24 h delay might produce such a facilitative effect. Indeed, a comparison of different VR schedules at a reactivation session on the day after training (albeit across different studies), indicates that there is an interplay between training-reactivation delay and the VR schedule employed. While VR5 did not successfully destabilize the instrumental sucrose memory here, a VR20 schedule was effective previously (Exton-McGuinness et al., 2014). Originally, we had hypothesized that the strength and influence of the context-reward memory might mean that a reactivation session could preferentially destabilize the context memory over the instrumental memory; and that it is possible for only one memory representation to be destabilized and its reconsolidation impaired at a time. While speculative, the greater reduction in context-reward association inherent in a VR20 session compared to a VR5 session (at least if conceptualized in terms of density of reward per unit time spent in the operant context) might lead to a preferential destabilization of the instrumental memory. Given that the initiation of destabilization appears to occur at session offset (Pedreira et al., 2004), the experience gained during the reactivation session can influence what memory, if any, is destabilized subsequently. Nevertheless, the failure of an interposed explicit context extinction session to facilitate the destabilizing effect of a VR5 reactivation may be somewhat problematic for this account. We had expected that context extinction would promote the destabilization of the instrumental memory at a subsequent VR5 reactivation session, due to the reduction in the expression of the context-reward association. However, it is possible that instead it introduced the influence of a context no-reward extinction memory, which itself might have been destabilized by the VR5 session. Indeed, extinction memory traces undergo destabilization and reconsolidation (Garcia-Delatorre et al., 2010; Rossato et al., 2010). In these studies, reminder of the training/extinction preferentially destabilized the extinction memory, such that amnestic treatment impaired the extinction memory to maintain subsequent memory expression (rather than impairing the original excitatory memory to reduce subsequent memory expression). It is also possible that the 30-min exposure to the training context did not result in appreciable context extinction in order to impact upon subsequent instrumental memory destabilization. We did not record behavior during the context extinction session; nor do our VR5 reactivation or test sessions have any behavioral measures that would be sensitive to the impact of context extinction.

The lack of effect of MK-801 when injected prior to either or both of two consecutive VR5 sessions is inconsistent with some accounts of our data. First, it is not simply the timing of the VR5 session being 48 h after the end of training that is important, as the interposition of another VR5 session rendered it ineffective in the Saline/MK-801 group. Second, it is unlikely that the VR5 session one day after training preferentially destabilized the only competing memory to the instrumental memory. If that were the case, we would have expected that MK-801 should have impaired the reconsolidation of that competing memory thereby allowing the subsequent VR5 session to destabilize the instrumental memory. MK-801 has been demonstrated to impair the reconsolidation of a wide range of memories (Milton and Everitt, 2010; Reichelt and Lee, 2013; Puaud et al., 2018; Bolsoni and Zuardi, 2019; Drame et al., 2020), and so it is equally unlikely that a preferentially destabilized memory would not have been disrupted by MK-801. Instead, it is possible that the interplay between different memory traces is not at the level of memory destabilization *per se*. Rather, the presence of a competing memory trace in itself might place a constraint on memory destabilization. This bears some similarity to the concept of trace dominance, which provides an explanation for the effect of amnestic treatment on reconsolidation vs. extinction depending on the parameters of memory reactivation (Eisenberg et al., 2003). However, it should be noted that in the present setting, there is not the parametric continuum that appears to distinguish memory destabilization from memory extinction (Suzuki et al., 2004; Flavell and Lee, 2013; Merlo et al., 2014).

Returning to the reason why delaying the VR5 reactivation session renders it more effective at destabilizing the instrumental memory, we sought to determine whether any critical active mnemonic process takes place during the course of the 48-h delay. Forgetting has been demonstrated to take place actively during the post-learning period, in a manner that is disrupted by the injection of memantine (Sachser et al., 2016). Moreover, the presence of any extended consolidation process extending up to 24 h after training (Taubenfeld et al., 2001; Katche et al., 2010), which could interact with destabilization, might also be disrupted by memantine. However, our results showed instead that the mere process of handling and injecting the rats in the control vehicle condition counteracted the facilitative effect of the extra day delay. Moreover, this counteracting effect appeared to involve some form of plasticity or memory, as memantine reinstated the destabilizing impact of the VR5 session. While we cannot fully account for these results, alongside the double reactivation experiment they do strongly reinforce the importance of there being an uninterrupted delay between the end of training and the VR5 reactivation session.

The interplay between training-reactivation delay and the degree of contingency change necessary to destabilize the instrumental memory may not, in fact, rely upon any other competing memory traces. The two best-characterized boundary conditions on memory reconsolidation are those of memory age and memory strength (Kida, 2020). In general, older and stronger memories are more resistant to destabilization (Milekic and Alberini, 2002; Suzuki et al., 2004; Wang et al., 2009). If we can assume that the necessity for a VR20 reactivation reflects greater resistance to destabilization one day, compared to 2 days after the end of training, it is perhaps more likely linked to the boundary condition of memory strength. For this to be valid, we would need to conclude that the instrumental memory decays to some extent over the course of 1–2 days after training has ended. However, within our data we cannot provide any supporting evidence for this (as behavior at the reactivation session is constrained by the maximum number of rewards gained, and also is influenced by the pre-session injection of MK-801). The extra day age of the instrumental memory is unlikely to be a factor as the boundary condition of memory age appears to emerge after weeks (Milekic and Alberini, 2002; Suzuki et al., 2004); equally, the re-emergence of memory destabilization of strongly learned fear memories occurs around 30 days (Wang et al., 2009).

In summary, the capacity of a specific memory reactivation experience to destabilize a well-learned instrumental memory is modulated by the delay between the end of training and the reactivation session. However, it remains unclear by what mechanism the delay facilitates, or indeed the lack of delay inhibits, memory destabilization. This is consistent with our current general lack of understanding over memory destabilization, the appropriate parameters to induce it, and the boundary conditions surrounding it (Jardine et al., 2022).

## Data availability statement

The raw data supporting the conclusions of this article will be made available by the authors, without undue reservation.

## Ethics statement

The animal study was reviewed and approved by University of Birmingham Animal Welfare and Ethical Review Board.

## Author contributions

CC and ME-M conducted the experiments. CC and JL analyzed the data and wrote the manuscript. All authors designed the experiments, contributed to the article, and approved the submitted version.
