# Supplementary material for: Procedures between training and reactivation influence the destabilization of instrumental sucrose memory
Source: Front Behav Neurosci. 2022 Sep 14;16:953629. doi: 10.3389/fnbeh.2022.953629 (PMC9524351; doi:10.3389/fnbeh.2022.953629)
Supplement: Supplementary file 1 [file Data_Sheet_1.docx]

**Pre-training context exposure**

Rats were pre-exposed to the training context for 30 min on the day before the start of instrumental training. VR5 memory reactivation took place on the day after the end of instrumental training. There were no differences in lever presses between the Saline and MK-801-treated groups at the test session (F(1,14)=0.008, p=0.929, η^2^_p_=0.001), reactivation session (F(1,14)=3.25, p=0.093, η^2^_p_=0.188) or during training (F(1,14)<0.001, p=0.988, η^2^_p_<0.001).


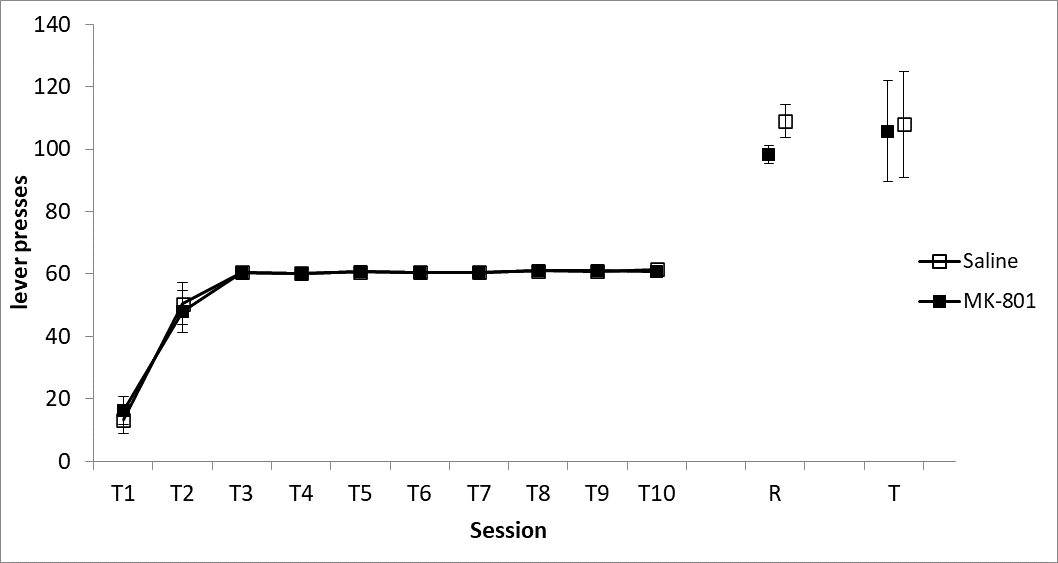


**Fig. S1.** No differences between Saline and MK-801-treated rats across any phase of the experiment when the 30-min context extinction session took place before the start of instrumental training. Data presented as mean ± SEM.

**Analyses of session length**

*Context extinction vs direct reactivation experiment*

There were no differences in session length between context extinction and direct reactivation groups at the reactivation session (Condition x MK-801: F(1,44)=0.67, p=0.417, η^2^_p_=0.015; MK-801: F(1,44)=3.57, p=0.066, η^2^_p_=0.075; context extinction condition: F(1,22)=0.85, p=0.367, η^2^_p_=0.037; direct reactivation condition: F(1,22)=2.76, p=0.111, η^2^_p_=0.112), or during training (Condition x MK-801: F(1,44)=0.06, p=0.804, η^2^_p_=0.001; MK-801 (F(1,44)=0.62, p=0.434, η^2^_p_=0.014; context extinction condition: F(1,22)=0.15, p=0.707, η^2^_p_=0.007; direct reactivation condition: F(1,22)=0.54, p=0.469, η^2^_p_=0.024).


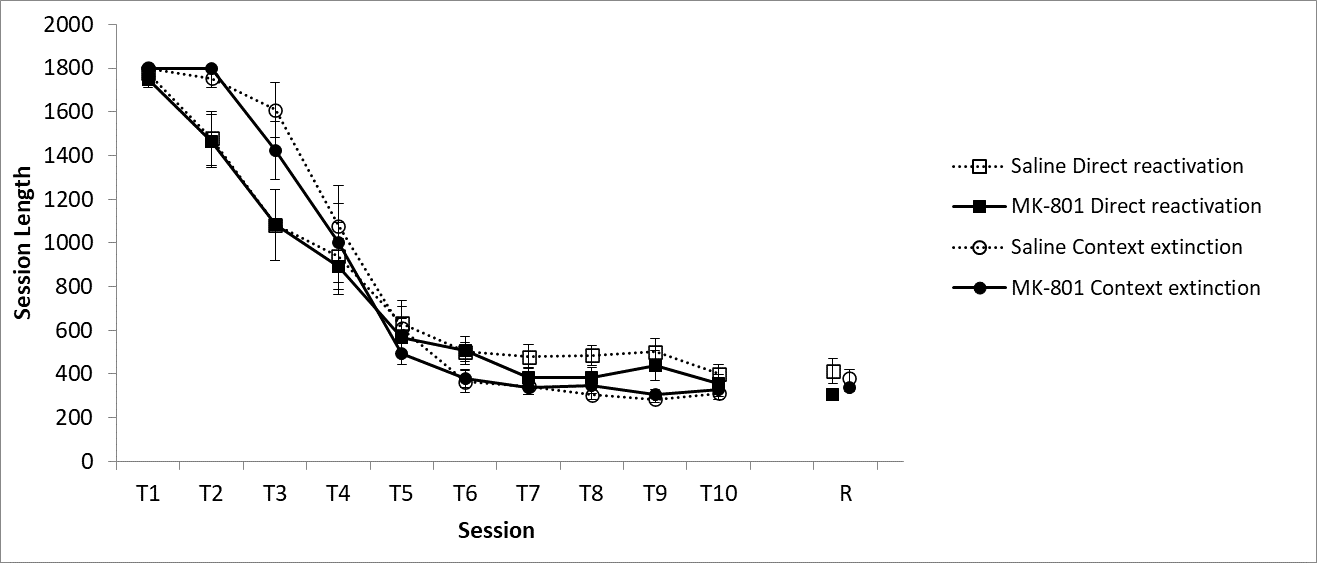


**Fig. S2.** In the comparison between the context extinction and direct reactivation conditions, there were no differences in session length between reactivation conditions and drug treatments across training or reactivation. Data presented as mean ± SEM.

*Context extinction vs delayed reactivation experiment*

There were no differences in session length between the context extinction and delayed reactivation groups at the reactivation session (Condition x MK-801: F(1,27)=1.29, p=0.266, η^2^_p_=0.046; MK-801: F(1,27)=2.16, p=0.153, η^2^_p_=0.074; context extinction condition: F(1,14)=0.05, p=0.819, η^2^_p_=0.004; delayed reactivation condition: F(1,13)=3.53, p=0.083, η^2^_p_=0.213), or during training (Condition x MK-801: F(1,27)=0.95, p=0.339, η^2^_p_=0.034; MK-801 (F(1,27)=0.31, p=0.584, η^2^_p_=0.011; context extinction condition: F(1,14)=0.11, p=0.748, η^2^_p_=0.008; delayed reactivation condition: F(1,13)=0.97, p=0.342, η^2^_p_=0.070).


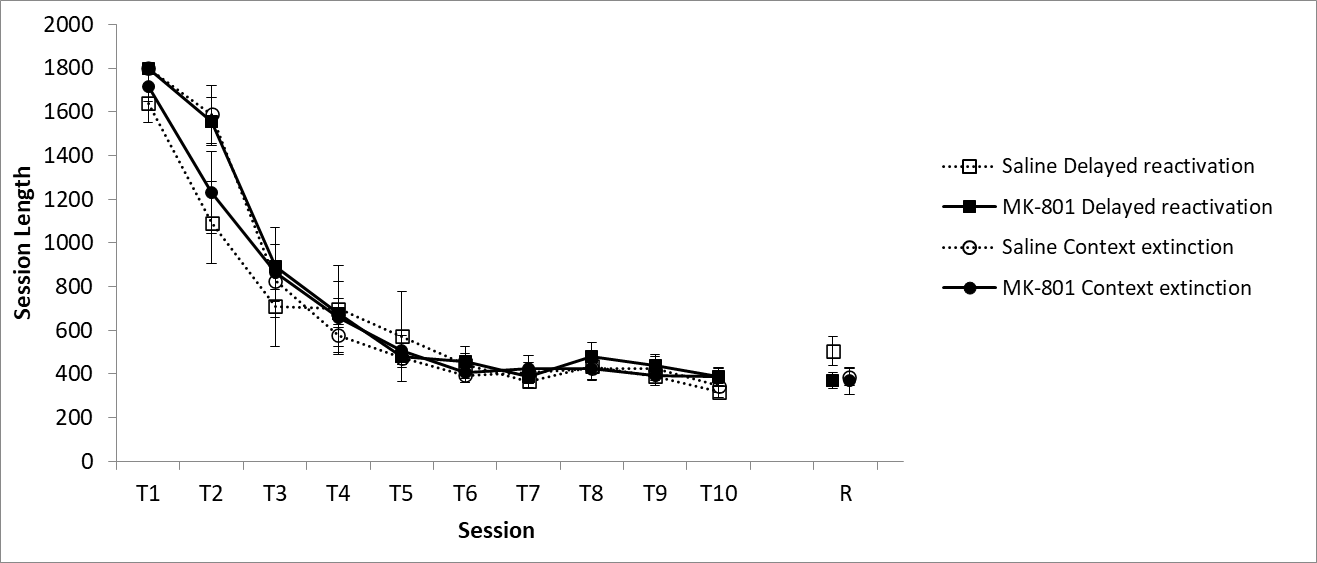


**Fig. S3.** In the comparison between the context extinction and delayed reactivation conditions, there were no differences in session length between reactivation conditions and drug treatments across training or reactivation. Data presented as mean ± SEM.

*Direct reactivation vs delayed reactivation experiment*

In the comparison of session lengths between the direct and delayed reactivation conditions, there was no condition x MK-801 interaction (F(1,28)=0.75, p=0.393, η^2^_p_=0.026) but there was a main effect of MK-801 (F(1,28)=6.71 p=0.015, η^2^_p_=0.193). Planned comparisons revealed that there was no effect of MK-801 in the direct reactivation condition (F(1,14)=1.53, p=0.236, η^2^_p_=0.099), but
MK-801-treated rats finished the reactivation session more rapidly than saline-treated rats in the delayed reactivation (F(1,14)=5.81, p=0.030, η^2^_p_=0.293). In contrast, during training there were no differences in session length (Condition x MK-801: F(1,28)=0.08, p=0.776, η^2^_p_=0.003; MK-801 (F(1,28)=0.20, p=0.662, η^2^_p_=0.007; direct reactivation condition: F(1,14)=0.18, p=0.681, η^2^_p_=0.012; delayed reactivation condition: F(1,14)=0.03, p=0.877, η^2^_p_=0.002).


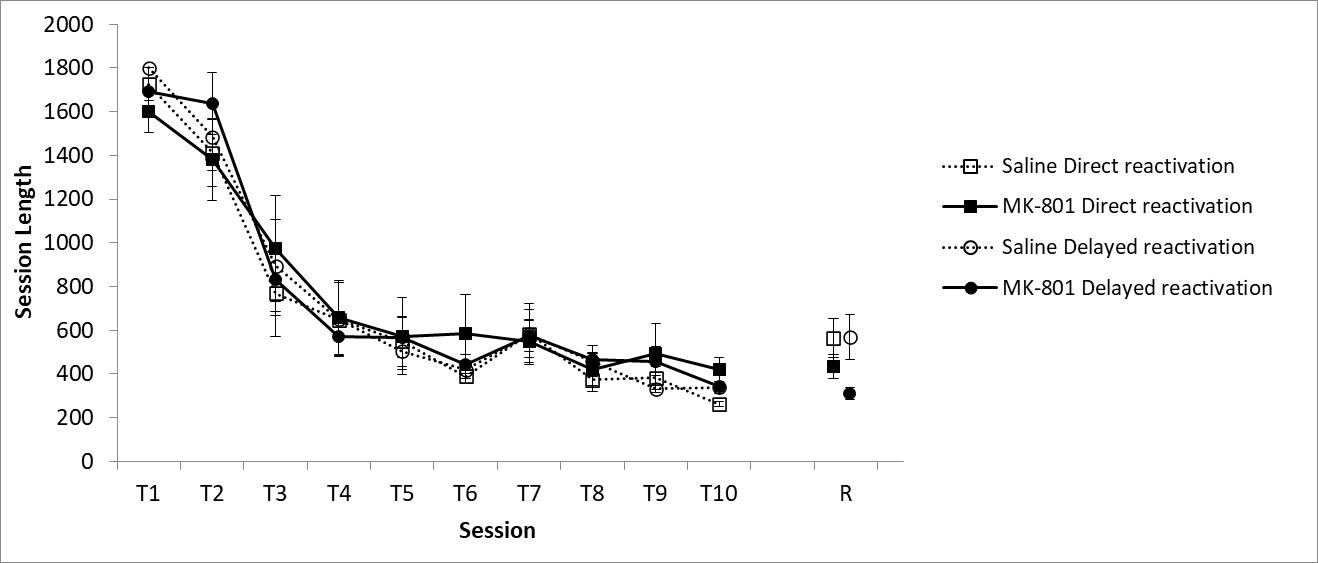


**Fig. S4.** In the comparison between the direct reactivation and delayed reactivation conditions, there were no differences in session length between reactivation conditions and drug treatments across training. However, there was an effect of MK-801 to reduce session length acutely at the reactivation session. This was more evident in the delayed reactivation condition. Data presented as mean ± SEM.
